# Hindfoot alignment at one year after total knee arthroplasty

**DOI:** 10.1007/s00167-015-3916-x

**Published:** 2015-12-24

**Authors:** Takashi Takenaka, Kazuya Ikoma, Suzuyo Ohashi, Yuji Arai, Yusuke Hara, Keiichiro Ueshima, Koushiro Sawada, Toshiharu Shirai, Hiroyoshi Fujiwara, Toshikazu Kubo

**Affiliations:** Department of Orthopaedics, Graduate School of Medical Science, Kyoto Prefectural University of Medicine, 465 Kajiicho, Kawaramachi-Hirokoji, Kamigyo-ku, Kyoto, 6028566 Japan

**Keywords:** Varus knee osteoarthritis, Total knee arthroplasty, Varus–valgus angle, Alignment compensation

## Abstract

**Purpose:**

It has previously been found that valgus hindfoot alignment (HFA) improves 3 weeks following total knee arthroplasty (TKA) for varus knee osteoarthritis (OA). In the present study, HFA was evaluated prior to TKA, as well as 3 weeks and 1 year following TKA. Using these multiple evaluations, the chronological effects of TKA on HFA were investigated.

**Methods:**

The study included 71 patients (73 legs) who underwent TKA for varus knee OA. Radiograph examinations of the entire limb and hindfoot were performed in the standing position prior to TKA, as well as 3 weeks and 1 year following TKA. The varus–valgus angle was used as an indicator of HFA in the coronal plane. Patients were divided into two groups according to the preoperative varus–valgus angle: a hindfoot varus group (varus–valgus angle <76°) and a hindfoot valgus group (varus–valgus angle ≥76°). The changes in the varus–valgus angle were evaluated and compared in both groups.

**Results:**

In the hindfoot valgus group, the mean ± standard deviation varus–valgus angle significantly declined from 80.5 ± 3.1° prior to TKA to 78.6 ± 3.7° 3 weeks following TKA and 77.1 ± 2.7° 1 year following TKA. However, in the hindfoot varus group, the mean varus–valgus angle prior to TKA (72.7 ± 2.6°) did not differ significantly from the mean varus–valgus angles 3 weeks (72.3 ± 3.3°) or 1 year (73.5 ± 3.0°) following TKA.

**Conclusions:**

HFA improved chronologically in legs with hindfoot valgus as a result of the alignment compensation ability of the hindfoot following TKA. However, no improvement was noted in legs with hindfoot varus because the alignment compensation ability of the hindfoot had been lost. The patients with hindfoot varus should be attended for ankle pain in the outpatient clinic after TKA.

**Level of evidence:**

III.

## Introduction

Osteoarthritis (OA) causes varus or valgus deformation in the knee joint, resulting in abnormal coronal plane alignment. Additionally, the subtalar joint compensates for this deformity [7], with valgus subtalar joint deformation occurring along with varus knee OA and varus subtalar joint deformation occurring along with valgus knee OA [4, 8]. Moreover, total knee arthroplasty (TKA), which is performed for the treatment of knee OA, involves correcting lower limb alignment with a focus on the knee joint, and some reports have mentioned that changes occur in the adjacent hindfoot alignment as well [1, 10].

The novel radiological method has previously been used to evaluate hindfoot alignment (varus–valgus angle, VVA) in patients with varus knee OA. Evaluations were performed both prior to and 3 weeks following TKA, and the associated findings were compared to investigate the effects of TKA on hindfoot alignment [5, 8]. Although VVA declined following TKA in the group with hindfoot valgus prior to surgery, no significant change was observed in VVA following TKA in the group with hindfoot varus prior to surgery. However, changes in hindfoot alignment were only assessed within the 3 weeks following TKA, which represents a fairly short period. It was hypothesized that improvements in both the hindfoot valgus and varus could be revealed by a 1-year follow-up after TKA had been used to correct extremity alignment, owing to the influences of load and dynamic movement. The present study was therefore concentrated on the 1-year follow-up. Specifically, the study was directed at assessing the chronological effects of TKA by evaluating and comparing hindfoot alignment before, 3 weeks after, and 1 year after the procedure had been performed. The chronological effects 1 year after TKA have not been understood and should be revealed because cases of hindfoot pain newly developing after TKA have been experienced, and it is considered that postoperative coronal alignment of the hindfoot was involved in this pain.

## Materials and methods

In the previous study, a total of 82 patients (100 legs) who underwent TKA for varus knee OA at Kyoto Prefectural University of Medicine between 2007 and 2011 were included, and they were evaluated at 3 weeks following surgery. The present study included 71 (73 legs) of these patients, each of whom underwent radiographic evaluation 1 year following surgery. Of the 11 patients who were excluded from the present study, two did not undergo radiographic evaluation of the hindfoot and nine did not participate in the radiographic evaluation at 1 year following surgery because they were being followed up at other hospitals. The mean age of the patients at the time of TKA was 74.7 (SD, 6.3) years, and of the 73 included legs, 17 belonged to male patients (mean age ± SD, 76.9 ± 6.8 years) and 56 belonged to female patients (mean age ± SD, 73.6 ± 6.1 years). Of the limbs, 32 were right legs and 41 were left legs. The exclusion criteria were knee deformities due to rheumatoid arthritis, posttraumatic knee OA, a history of surgery on the affected side (such as high tibial osteotomy), and revision surgery.

### Surgical method

The implants used were posterior stabilized-type models, and included Zimmer NexGen LPS-FLEX mobile bearing (*n* = 40; Zimmer, Warsaw, IN, USA), Zimmer NexGen LPS-FLEX fixed bearing (*n* = 9; Zimmer), and Stryker Scorpio NRG (*n* = 24; Stryker Howmedica Osteonics, NJ, USA), with the femoral, tibial, and patellar components fixed using cement. Surgery was performed using the measured resection method.

### Radiographic examination

**Fig. 1:**
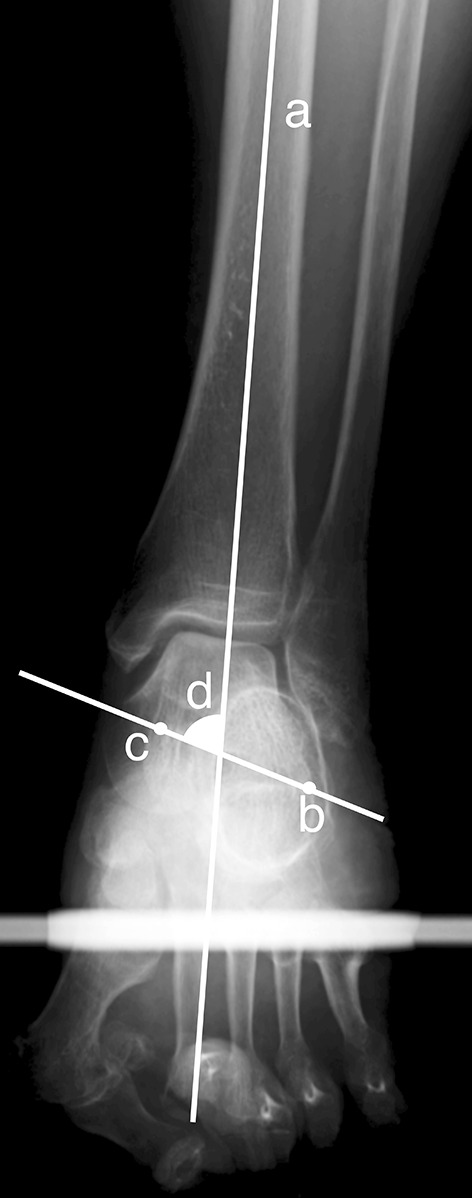
Hindfoot radiograph image. *Line* *a* indicates the long axis of the tibia. *Point* *b* indicates the lateral extremity of the calcaneus at the posterior surface of the talocalcaneal joint. *Point* *c* indicates the superior margin of the sustentaculum tali. *Angle* *d* is the varus–valgus angle

Hindfoot radiographic examination was performed prior to TKA, 3 weeks following TKA, and 1 year following TKA. A specially designed foot stand that forces the hindfoot to be flat and the forefoot plantar to be flexed at 30° from the metatarsal region was used for radiographic examination. The patient was instructed to stand with both legs on the specially designed foot stand, and a radiograph film was placed vertically against the toes. Radiographs were radiated from a horizontal plane with inclination of the dorsal side by 5°. Both hindfeet were imaged with an imaging distance of 120 cm. On the radiograph image of the hindfoot, hindfoot alignment was evaluated by measuring the angle between the long axis of the tibia and the line connecting the superior margin of the sustentaculum tali, which was determined from the imaging of the hindfoot to the lateral extremity of the calcaneus at the posterior surface of the talocalcaneal joint (VVA). The VVA was used to indicate hindfoot alignment in the coronal plane (Fig. 1) [8]. Additionally, radiographic examination of the entire limb was performed in the standing position prior to and 3 weeks following TKA, and the femorotibial angle (FTA) was used as an indicator of lower limb alignment in the coronal plane [4, 12]. Measurements were taken by two orthopaedic specialists (board-certified orthopaedic surgeons) using measurement software (ImageJ; National Institutes of Health, Bethesda, MD, USA) and calculated to one decimal place. Based on the findings of a previous study, 76.0° was used as the threshold for hindfoot varus and valgus because this value is the average hindfoot angle in the Asian population [8]. The included cases were divided into two groups according to the preoperative VVA: a hindfoot varus group (VVA < 76.0°) and hindfoot valgus group (VVA ≥ 76.0°). The changes in the VVA were evaluated and compared in both groups.

The study was approved by the medical ethics review board of Kyoto Prefectural University of Medicine (application number: ERB-C-114), and informed consent was obtained from all patients in the study.

### Statistical analysis

A paired *t* test was used to evaluate the FTA prior to and following TKA, and the chronological changes in the VVA were investigated using repeated analysis of variance with Tukey’s post hoc test. Total sample size (*n* = 24) provided 80 % or greater power to detect a 25 % difference in VVA between any two groups (*α* = 0.05, *β* = 0.20). All statistical analyses were performed using SPSS for Windows, version 20.0 (IBM Corp., Armonk, NY, USA), and a *p* value <0.05 was considered to indicate a significant difference. Continuous variables were summarized in terms of their means ± their standard deviations.

## Results

The hindfoot valgus group included 51 legs (22 right and 29 left legs). In comparison, the hindfoot varus group included 22 legs (9 right and 13 left legs) (Table 1).

**Table 1:** Patients’ characteristics

	Valgus group (*n* = 51)	Varus group (*n* = 22)	*p* value
Age (year)	74.7 ± 6.7	75.0 ± 5.5	
Sex			
	Male: 8	Male: 7	
	Female: 43	Female: 15	
FTA			
Pre-op	185.5 ± 4.5°	183.0 ± 5.1°	0.04
Post-op	173.9 ± 2.8°	174.0 ± 2.7°	n.s.

*FTA* femorotibial angle

In the 3 weeks following TKA, the mean FTA significantly declined in both the hindfoot valgus group (*p* < 0.001) and the hindfoot varus group (*p* < 0.001). Prior to TKA, the mean FTA was significantly higher in the hindfoot valgus group than in the hindfoot varus group (*p* = 0.04), but the groups did not differ significantly 3 weeks after TKA (n.s.) (Table 2).

**Table 2:** Femorotibial angle prior to and 3 weeks following total knee arthroplasty

Group	Preoperation	Postoperation	*p* value
Valgus group	185.5 ± 4.5°	173.9 ± 2.8°	<0.001
Varus group	183.0 ± 5.1°	174.0 ± 2.7°	<0.001

Data are presented as mean ± SD

*TKA* total knee arthroplasty, *FTA* femorotibial angle

In the hindfoot valgus group, the mean VVA significantly declined from 80.5 ± 3.1° prior to TKA to 78.6 ± 3.7° (*p* < 0.01) 3 weeks following surgery and to 77.1 ± 2.7° (*p* < 0.05) 1 year following surgery (Table 3). However, in the hindfoot varus group, the mean VVA prior to TKA (72.7 ± 2.6°) did not differ significantly from the mean VVAs 3 weeks (72.3 ± 3.3°; n.s.) or 1 year (73.5 ± 3.0°; n.s.) following surgery (Table 3).

**Table 3:** Varus–valgus angle before, 3 weeks after, and 1 year after total knee arthroplasty

	VVA (°)		
	Prior to TKA	3 weeks following TKA	1 year following TKA
Hindfoot valgus group	80.5 ± 3.1	78.6 ± 3.7**	77.1 ± 2.7*
Hindfoot varus group	72.7 ± 2.6	72.3 ± 3.3^n.s.^	73.5 ± 3.0^n.s.^

Data are presented as mean ± SD

Chronological changes in the VVA were investigated using repeated analysis of variance with Tukey’s post hoc test

*TKA* total knee arthroplasty, *VVA* varus–valgus angle, *n.s.* not significant

* *p* < 0.05; ** *p* < 0.01

## Discussion

The most important finding of the present study was that, in the hindfoot valgus group, hindfoot alignment had improved 3 weeks after TKA, and further improvement was noted 1 year following TKA. These findings indicate that alignment compensation ability had been maintained in the hindfoot valgus group. However, in the hindfoot varus group, no such improvements were noted at 3 weeks or 1 year following TKA. These findings indicate that alignment compensation ability had been lost in the hindfoot varus group.

Mullaji et al. [10] have reported a relationship between varus knee deformity and hindfoot valgus. Moreover, Norton et al. [11] investigated 401 knees of 324 patients with knee OA and reported both a relationship between varus knee deformity and hindfoot valgus and relationships between valgus and varus knee deformities and hindfoot varus. Additionally, the authors observed that a varus knee deformity would result in a hindfoot valgus of 0.5°, while a valgus knee deformity would result in a hindfoot varus of 0.4°. However, Chandler et al. [1] reported no relationship between deformities due to knee OA and deformities of the hindfoot. There is currently no consensus regarding the effects of knee deformities on hindfoot alignment. Moreover, TKA involves the correction of lower limb alignment, and changes are believed to occur in hindfoot alignment. However, few reports have been published regarding changes in hindfoot alignment following TKA, and limited information is available. Chandler et al. [1] reported an approximately 50 % decline in the varus and valgus angles of the hindfoot following TKA. Further, Mullaji et al. [10] reported a decline in the valgus angle of the hindfoot following TKA.

In the previous study, the evaluation was limited to varus knee OA and the investigation was confined to hindfoot alignment prior to and immediately following TKA. Prior to TKA, subtalar joint valgus and varus were observed in 70 and 30 % of varus knee OA cases, respectively. Although the hindfoot valgus improved immediately following TKA, hindfoot varus did not show any improvement following TKA [5]. Regarding coronal plane alignment of the lower limb with the femur head as the point of origin, compensation for a change in the load axis of the lower limb can only be achieved with subtalar joint movement in the coronal plane. Similarly, hindfoot valgus in varus knee OA is believed to result from the compensation of the hindfoot for the varus knee deformity, as has been reported previously [10, 11]. However, the alignment compensation ability of the hindfoot with hindfoot varus is lost in varus knee OA.

In the present study, the investigation focused on examining change in hindfoot alignment 1 year following TKA. The previous study showed that TKA resulted in alignment correction of the lower limbs. It was hypothesized that improvements in hindfoot alignment could be associated with the dynamic stresses that are associated with the activities of daily living (such as walking) in addition to the load immediately after the operation. Consistent with this hypothesis, it was observed that hindfoot valgus was even closer to 76°. Long-term improvements are also expected to result from the dynamic movements in the hindfoot valgus that are associated with increased flexibility. However, contrary to the hypothesis, there was no improvement in the hindfoot varus group. Even with dynamic stress in the rigid hindfoot varus, improvements were not observed in 1 year. In the future, a study with additional follow-up may reveal whether there are long-term improvements in the hindfoot varus group.

When medial deviation of the lower limb load axis occurs because of varus knee OA, the subtalar joint compensates for the deviation with valgus movement. However, if the medial deviation of the load axis becomes large and the subtalar joint is not able to compensate, valgus movement changes to varus movement. Extended movement is believed to result in a rigid varus subtalar joint without the ability to compensate; because the ability to compensate has been lost, the joint remains varus after TKA. The presence of a rigid varus subtalar joint is concerning because of the high possibility of disorders resulting from stress concentration on the medial side of the adjacent ankle after TKA. Additionally, if the FTA is overcorrected, the occurrence of disorders resulting from concentration on the lateral side of the adjacent ankle is also concerning. Therefore, careful post-TKA follow-up is needed in patients with a varus subtalar joint prior to TKA.

In clinical practice, some patients report pain in the foot on the treated side following TKA. Such pain is believed to occur in cases with continued hindfoot varus. Because the presence of pain in the hindfoot was not investigated in the present study, this topic can only be speculated on by the authors. Nevertheless, coronal plane alignment of the lower limb is believed to have improved because TKA adversely affects the hindfoot fixed in an inverted position, thereby causing pain. In the future, confirmation of the presence of pain in the hindfoot prior to and following TKA, determination of the area of pain, and the development of measures to overcome pain following TKA will be necessary.

This study has several limitations. First, Cobey’s method or other conventional measurement methods were not used [2, 3, 6, 9, 13, 14]. In Cobey’s method, the bone axis of the calcaneus is used to measure the angle of the major axis of the tibia [2, 3]. However, determination of the calcaneal axis is uncertain in this method, resulting in accidental errors in measurement. In Saltzman’s method, hindfoot alignment is indicated by the distance between the contact area and the tibial axis [13]. In the present study, our novel hindfoot measurement method [8] was employed under the belief that the varus–valgus angle is important for measuring hindfoot alignment; however, the relationship between our method and Cobey’s method should be investigated in future studies. Second, the number of hindfoot varus cases was small. Of the 73 limbs included in this study, hindfoot varus was present in only 22 limbs.

The clinical relevance of this study is that the patients with hindfoot varus before TKA may be taken care of because they may complain the hindfoot pain newly developing after TKA.

## Conclusion

In conclusion, hindfoot alignment improved chronologically in legs with hindfoot valgus as a result of the alignment compensation ability of the hindfoot following TKA. However, no improvement was noted in legs with hindfoot varus because the alignment compensation ability of the hindfoot had been lost. The patients with hindfoot varus should be attended for ankle pain in the outpatient clinic after TKA.
